# 1,10-Phenanthroline–dithio­oxamide (2/1)

**DOI:** 10.1107/S1600536810016405

**Published:** 2010-05-12

**Authors:** Hoong-Kun Fun, Wan-Sin Loh, Annada C. Maity, Shyamaprosad Goswami

**Affiliations:** a X-ray Crystallography Unit, School of Physics, Universiti Sains Malaysia, 11800 USM, Penang, Malaysia; b Department of Chemistry, Bengal Engineering and Science University, Shibpur, Howrah 711 103, India

## Abstract

The asymmetric unit of the title compound, C_12_H_8_N_2_·0.5C_2_H_4_N_2_S_2_, contains one 1,10-phenanthroline mol­ecule and a half-mol­ecule of dithio­oxamide, which lies across a crystallographic inversion center. The 1,10-phenanthroline unit is not strictly planar, with dihedral angles between the central benzene ring and the pyridine rings of 1.42 (10) and 1.40 (10)°. In the crystal structure, two 1,10-phenanthroline mol­ecules are linked together by one dithio­oxamide *via* inter­molecular N—H⋯N hydrogen bonds.

## Related literature

For background to the chemistry of 1,10-phenanthroline, see: Goswami *et al.* (2005 ▶); Han *et al.* (2009 ▶); Ishida *et al.* (2010 ▶). For a related structure, see: Fun *et al.* (2010 ▶). For standard bond-length data, see: Allen *et al.* (1987 ▶). For the stability of the temperature controller used for the data collection, see: Cosier & Glazer (1986 ▶).
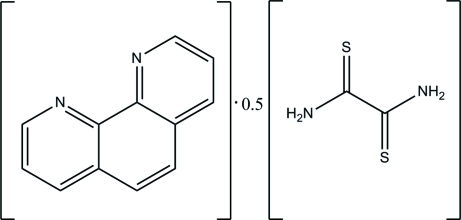


## Experimental

### 

#### Crystal data

C_12_H_8_N_2_·0.5C_2_H_4_N_2_S_2_
*M* *_r_* = 240.30
Monoclinic, 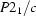

*a* = 10.5481 (3) Å
*b* = 10.0544 (3) Å
*c* = 13.9960 (4) Å
β = 130.145 (2)°
*V* = 1134.65 (6) Å^3^
*Z* = 4
Mo *K*α radiation
μ = 0.26 mm^−1^
*T* = 100 K
0.28 × 0.26 × 0.10 mm

#### Data collection

Bruker SMART APEXII CCD area-detector diffractometer
Absorption correction: multi-scan (*SADABS*; Bruker, 2009 ▶) *T*_min_ = 0.931, *T*_max_ = 0.974
22512 measured reflections
3374 independent reflections
2307 reflections with *I* > 2σ(*I*)
*R*_int_ = 0.074

#### Refinement

*R*[*F* ^2^ > 2σ(*F* ^2^)] = 0.062
*wR*(*F* ^2^) = 0.160
*S* = 1.07
3374 reflections
162 parameters
H atoms treated by a mixture of independent and constrained refinement
Δρ_max_ = 1.24 e Å^−3^
Δρ_min_ = −0.40 e Å^−3^

### 

Data collection: *APEX2* (Bruker, 2009 ▶); cell refinement: *SAINT* (Bruker, 2009 ▶); data reduction: *SAINT*; program(s) used to solve structure: *SHELXTL* (Sheldrick, 2008 ▶); program(s) used to refine structure: *SHELXTL*; molecular graphics: *SHELXTL*; software used to prepare material for publication: *SHELXTL* and *PLATON* (Spek, 2009 ▶).

## Supplementary Material

Crystal structure: contains datablocks global, I. DOI: 10.1107/S1600536810016405/lh5039sup1.cif

Structure factors: contains datablocks I. DOI: 10.1107/S1600536810016405/lh5039Isup2.hkl

Additional supplementary materials:  crystallographic information; 3D view; checkCIF report

## Figures and Tables

**Table 1:** Hydrogen-bond geometry (Å, °)

*D*—H⋯*A*	*D*—H	H⋯*A*	*D*⋯*A*	*D*—H⋯*A*
N3—H3*C*⋯N1^i^	0.81 (3)	2.08 (3)	2.876 (3)	167 (4)

Symmetry code: (i) 
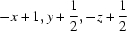
.

**Fig. 1.**
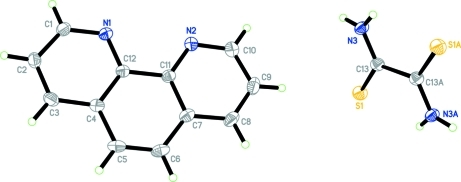
The molecular structure of the title compound, showing 50% probability displacement ellipsoids and the atom-numbering scheme. Atoms with suffix A [S1A, C13A and N3A] were generated by symmetry code -x+2, -y+2, -z+1.

**Fig. 2.**
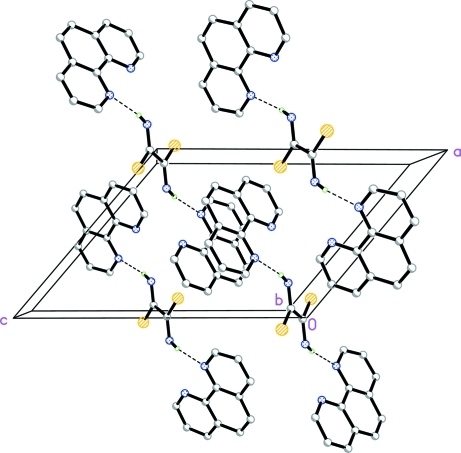
The crystal packing of the title compound, viewed along the a axis. H atoms not involved in the intermolecular interactions (dashed lines) have been omitted for clarity.

## supplementary crystallographic information

### Comment

1,10-Phenanthroline plays a very important role in the field of molecular recognition and supramolecular chemistry (Goswami *et al.*, 2005). We have used 1,10-phenanthroline in the recognition of urea by designed synthetic receptors (Goswami *et al.*, 2005). The title compound is also used in supramolecular co-ordination chemistry (Ishida *et al.*, 2010; Han *et al.*, 2009). Here we report the co-crystal of the 1,10-phenanthroline and guest molecule dithiooxamide.

The asymmetric unit (Fig. 1), consists of one 1,10-phenanthroline and a half dithiooxamide. The dithiooxamide lies across a crystallographic inversion center [symmetry code = -x+2, -y+2, -z+1]. The 1,10-phenanthroline unit is not strictly planar, with dihedral angles between the central ring and the C1–C4/C12/N1 and C7–C10/N2/C11 rings of 1.42 (10) and 1.40 (10)°, respectively. The bond lengths are within normal ranges (Allen *et al.*, 1987) and are comparable to those observed for closely related structure (Fun *et al.*, 2010).

In the crystal structure (Fig. 2), two 1,10-phenanthroline molecules are linked together by one dithiooxamide *via* intermolecular N3—H3C···N1(-x+1, y+1/2, -z+1/2)   hydrogen bonds (Table 1).

### Experimental

A mixture of commercially available 1,10-phenanthroline and dithiooxamide (1:1) was dissolved in methanol-chloroform (v/v 2:1). Single crystals were grown by slow evaporation of the solvent.

### Refinement

H3B and H3C were located in a difference Fourier map and refined freely [N–H = 0.82 (2) and 0.85 (2) Å]. The remaining H atoms were positioned geometrically and refined using a riding model with *U*_iso_(H) = 1.2 *U*_eq_(C) [C–H = 0.93 Å]. In the final difference Fourier map, the highest peak and the deepest hole are 0.77 and 0.60 Å, respectively, from atom S1.

### Crystal data

**Table:**

C_12_H_8_N_2_·0.5C_2_H_4_N_2_S_2_	*F*(000) = 500
*M**_r_* = 240.30	*D*_x_ = 1.407 Mg m^−^^3^
Monoclinic, *P*2_1_/*c*	Mo *K*α radiation, λ = 0.71073 Å
Hall symbol: -P 2ybc	Cell parameters from 5115 reflections
*a* = 10.5481 (3) Å	θ = 2.5–30.1°
*b* = 10.0544 (3) Å	µ = 0.26 mm^−^^1^
*c* = 13.9960 (4) Å	*T* = 100 K
β = 130.145 (2)°	Block, orange
*V* = 1134.65 (6) Å^3^	0.28 × 0.26 × 0.10 mm
*Z* = 4	

### Data collection

**Table:**

Bruker SMART APEXII CCD area-detector diffractometer	3374 independent reflections
Radiation source: fine-focus sealed tube	2307 reflections with *I* > 2σ(*I*)
graphite	*R*_int_ = 0.074
φ and ω scans	θ_max_ = 30.3°, θ_min_ = 2.5°
Absorption correction: multi-scan (*SADABS*; Bruker, 2009)	*h* = −14→14
*T*_min_ = 0.931, *T*_max_ = 0.974	*k* = −14→13
22512 measured reflections	*l* = −19→19

### Refinement

**Table:**

Refinement on *F*^2^	Primary atom site location: structure-invariant direct methods
Least-squares matrix: full	Secondary atom site location: difference Fourier map
*R*[*F*^2^ > 2σ(*F*^2^)] = 0.062	Hydrogen site location: inferred from neighbouring sites
*wR*(*F*^2^) = 0.160	H atoms treated by a mixture of independent and constrained refinement
*S* = 1.07	*w* = 1/[σ^2^(*F*_o_^2^) + (0.0764*P*)^2^ + 0.8154*P*] where *P* = (*F*_o_^2^ + 2*F*_c_^2^)/3
3374 reflections	(Δ/σ)_max_ < 0.001
162 parameters	Δρ_max_ = 1.24 e Å^−^^3^
0 restraints	Δρ_min_ = −0.40 e Å^−^^3^

### Special details

**Table:**

Experimental. The crystal was placed in the cold stream of an Oxford Cryosystems Cobra open-flow nitrogen cryostat (Cosier & Glazer, 1986) operating at 100.0 (1) K.
Geometry. All esds (except the esd in the dihedral angle between two l.s. planes) are estimated using the full covariance matrix. The cell esds are taken into account individually in the estimation of esds in distances, angles and torsion angles; correlations between esds in cell parameters are only used when they are defined by crystal symmetry. An approximate (isotropic) treatment of cell esds is used for estimating esds involving l.s. planes.
Refinement. Refinement of *F*^2^ against ALL reflections. The weighted *R*-factor wR and goodness of fit *S* are based on *F*^2^, conventional *R*-factors *R* are based on *F*, with *F* set to zero for negative *F*^2^. The threshold expression of *F*^2^ > σ(*F*^2^) is used only for calculating *R*-factors(gt) etc. and is not relevant to the choice of reflections for refinement. *R*-factors based on *F*^2^ are statistically about twice as large as those based on *F*, and *R*- factors based on ALL data will be even larger.

### Fractional atomic coordinates and isotropic or equivalent isotropic displacement parameters (Å^2^)

**Table:**

	*x*	*y*	*z*	*U*_iso_*/*U*_eq_	
N1	0.3548 (2)	0.2611 (2)	0.34019 (17)	0.0185 (4)	
N2	0.5551 (2)	0.4592 (2)	0.36522 (18)	0.0217 (4)	
N3	0.8022 (2)	0.9404 (2)	0.36819 (19)	0.0201 (4)	
C1	0.2582 (3)	0.1675 (2)	0.3304 (2)	0.0217 (5)	
H1A	0.1591	0.1482	0.2512	0.026*	
C2	0.2959 (3)	0.0962 (2)	0.4317 (2)	0.0234 (5)	
H2A	0.2241	0.0313	0.4198	0.028*	
C3	0.4421 (3)	0.1243 (3)	0.5494 (2)	0.0247 (5)	
H3A	0.4698	0.0791	0.6186	0.030*	
C4	0.5493 (3)	0.2219 (2)	0.5643 (2)	0.0193 (5)	
C5	0.7051 (3)	0.2534 (3)	0.6845 (2)	0.0240 (5)	
H5A	0.7382	0.2072	0.7549	0.029*	
C6	0.8035 (3)	0.3489 (3)	0.6961 (2)	0.0236 (5)	
H6A	0.9044	0.3670	0.7746	0.028*	
C7	0.7561 (3)	0.4235 (2)	0.5902 (2)	0.0193 (5)	
C8	0.8544 (3)	0.5272 (3)	0.6009 (2)	0.0248 (5)	
H8A	0.9528	0.5508	0.6790	0.030*	
C9	0.8045 (3)	0.5928 (3)	0.4964 (3)	0.0277 (6)	
H9A	0.8688	0.6604	0.5015	0.033*	
C10	0.6528 (3)	0.5553 (3)	0.3803 (2)	0.0267 (5)	
H10A	0.6190	0.6011	0.3095	0.032*	
C11	0.6054 (3)	0.3938 (2)	0.4689 (2)	0.0182 (5)	
C12	0.4998 (3)	0.2896 (2)	0.4565 (2)	0.0170 (4)	
C13	0.9644 (3)	0.9392 (2)	0.4567 (2)	0.0196 (5)	
S1	1.08871 (7)	0.81821 (6)	0.47638 (6)	0.02354 (18)	
H3C	0.752 (4)	0.884 (3)	0.314 (3)	0.030 (8)*	
H3B	0.754 (4)	1.004 (3)	0.366 (3)	0.032 (8)*	

### Atomic displacement parameters (Å^2^)

**Table:**

	*U*^11^	*U*^22^	*U*^33^	*U*^12^	*U*^13^	*U*^23^
N1	0.0163 (8)	0.0198 (10)	0.0163 (9)	−0.0001 (7)	0.0091 (7)	−0.0012 (7)
N2	0.0213 (9)	0.0205 (10)	0.0208 (9)	−0.0015 (8)	0.0125 (8)	0.0007 (8)
N3	0.0170 (9)	0.0203 (11)	0.0173 (9)	−0.0006 (8)	0.0085 (8)	−0.0037 (8)
C1	0.0182 (9)	0.0207 (12)	0.0216 (11)	−0.0002 (9)	0.0108 (9)	−0.0009 (9)
C2	0.0224 (10)	0.0200 (12)	0.0290 (12)	−0.0002 (9)	0.0171 (10)	0.0038 (10)
C3	0.0234 (11)	0.0260 (13)	0.0237 (12)	0.0047 (9)	0.0148 (10)	0.0081 (10)
C4	0.0183 (9)	0.0211 (12)	0.0172 (10)	0.0025 (8)	0.0108 (8)	0.0014 (9)
C5	0.0203 (10)	0.0314 (14)	0.0149 (10)	0.0062 (9)	0.0090 (9)	0.0033 (10)
C6	0.0184 (10)	0.0304 (14)	0.0152 (10)	0.0028 (9)	0.0078 (9)	−0.0042 (9)
C7	0.0154 (9)	0.0196 (12)	0.0200 (11)	0.0023 (8)	0.0101 (8)	−0.0039 (9)
C8	0.0147 (9)	0.0235 (13)	0.0285 (12)	−0.0012 (8)	0.0104 (9)	−0.0049 (10)
C9	0.0230 (11)	0.0205 (13)	0.0401 (15)	−0.0037 (9)	0.0206 (11)	−0.0027 (11)
C10	0.0255 (11)	0.0250 (13)	0.0280 (12)	−0.0015 (10)	0.0166 (10)	0.0028 (10)
C11	0.0165 (9)	0.0186 (12)	0.0177 (10)	0.0012 (8)	0.0103 (8)	−0.0010 (9)
C12	0.0172 (9)	0.0160 (11)	0.0164 (10)	0.0021 (8)	0.0102 (8)	−0.0007 (8)
C13	0.0205 (10)	0.0239 (13)	0.0157 (10)	−0.0008 (9)	0.0122 (9)	0.0015 (9)
S1	0.0235 (3)	0.0213 (3)	0.0248 (3)	0.0033 (2)	0.0151 (2)	−0.0009 (2)

### Geometric parameters (Å, °)

**Table:**

N1—C1	1.328 (3)	C5—C6	1.344 (4)
N1—C12	1.364 (3)	C5—H5A	0.9300
N2—C10	1.326 (3)	C6—C7	1.433 (3)
N2—C11	1.352 (3)	C6—H6A	0.9300
N3—C13	1.315 (3)	C7—C8	1.408 (3)
N3—H3C	0.81 (3)	C7—C11	1.420 (3)
N3—H3B	0.81 (3)	C8—C9	1.364 (4)
C1—C2	1.398 (3)	C8—H8A	0.9300
C1—H1A	0.9300	C9—C10	1.411 (3)
C2—C3	1.377 (3)	C9—H9A	0.9300
C2—H2A	0.9300	C10—H10A	0.9300
C3—C4	1.407 (3)	C11—C12	1.456 (3)
C3—H3A	0.9300	C13—C13^i^	1.535 (5)
C4—C12	1.412 (3)	C13—S1	1.676 (2)
C4—C5	1.438 (3)		
			
C1—N1—C12	117.8 (2)	C7—C6—H6A	119.3
C10—N2—C11	117.2 (2)	C8—C7—C11	117.5 (2)
C13—N3—H3C	123 (2)	C8—C7—C6	122.4 (2)
C13—N3—H3B	117 (2)	C11—C7—C6	120.1 (2)
H3C—N3—H3B	120 (3)	C9—C8—C7	119.7 (2)
N1—C1—C2	124.2 (2)	C9—C8—H8A	120.1
N1—C1—H1A	117.9	C7—C8—H8A	120.1
C2—C1—H1A	117.9	C8—C9—C10	118.2 (2)
C3—C2—C1	118.3 (2)	C8—C9—H9A	120.9
C3—C2—H2A	120.8	C10—C9—H9A	120.9
C1—C2—H2A	120.8	N2—C10—C9	124.5 (2)
C2—C3—C4	119.6 (2)	N2—C10—H10A	117.8
C2—C3—H3A	120.2	C9—C10—H10A	117.8
C4—C3—H3A	120.2	N2—C11—C7	122.9 (2)
C3—C4—C12	118.0 (2)	N2—C11—C12	118.79 (19)
C3—C4—C5	122.0 (2)	C7—C11—C12	118.3 (2)
C12—C4—C5	120.0 (2)	N1—C12—C4	122.1 (2)
C6—C5—C4	120.7 (2)	N1—C12—C11	118.4 (2)
C6—C5—H5A	119.7	C4—C12—C11	119.46 (19)
C4—C5—H5A	119.7	N3—C13—C13^i^	114.4 (3)
C5—C6—C7	121.4 (2)	N3—C13—S1	124.50 (19)
C5—C6—H6A	119.3	C13^i^—C13—S1	121.1 (2)
			
C12—N1—C1—C2	−0.2 (3)	C10—N2—C11—C12	178.7 (2)
N1—C1—C2—C3	0.2 (4)	C8—C7—C11—N2	0.9 (3)
C1—C2—C3—C4	−0.7 (4)	C6—C7—C11—N2	−179.0 (2)
C2—C3—C4—C12	1.3 (4)	C8—C7—C11—C12	−178.2 (2)
C2—C3—C4—C5	−178.6 (2)	C6—C7—C11—C12	1.9 (3)
C3—C4—C5—C6	−178.7 (2)	C1—N1—C12—C4	0.8 (3)
C12—C4—C5—C6	1.4 (4)	C1—N1—C12—C11	−178.8 (2)
C4—C5—C6—C7	0.8 (4)	C3—C4—C12—N1	−1.3 (3)
C5—C6—C7—C8	177.7 (2)	C5—C4—C12—N1	178.6 (2)
C5—C6—C7—C11	−2.4 (3)	C3—C4—C12—C11	178.2 (2)
C11—C7—C8—C9	−1.3 (3)	C5—C4—C12—C11	−1.8 (3)
C6—C7—C8—C9	178.6 (2)	N2—C11—C12—N1	0.7 (3)
C7—C8—C9—C10	1.2 (4)	C7—C11—C12—N1	179.81 (19)
C11—N2—C10—C9	0.3 (4)	N2—C11—C12—C4	−178.9 (2)
C8—C9—C10—N2	−0.7 (4)	C7—C11—C12—C4	0.2 (3)
C10—N2—C11—C7	−0.4 (3)		

Symmetry codes: (i) −*x*+2, −*y*+2, −*z*+1.

### Hydrogen-bond geometry (Å, °)

**Table:**

*D*—H···*A*	*D*—H	H···*A*	*D*···*A*	*D*—H···*A*
N3—H3C···N1^ii^	0.81 (3)	2.08 (3)	2.876 (3)	167 (4)

Symmetry codes: (ii) −*x*+1, *y*+1/2, −*z*+1/2.
